# Circulatory Management of Polymer Waste: Recycling into Fine Fibers and Their Applications

**DOI:** 10.3390/ma14164694

**Published:** 2021-08-20

**Authors:** Alena Opálková Šišková, Petra Peer, Anita Eckstein Andicsová, Igor Jordanov, Piotr Rychter

**Affiliations:** 1 Institute of Materials and Machine Mechanics, Slovak Academy of Sciences, Dúbravská cesta 9, 845 13 Bratislava, Slovakia; 2 Polymer Institute of Slovak Academy of Sciences, Dúbravská cesta 9, 845 41 Bratislava, Slovakia; anita.andicsova@savba.sk; 3 Institute of Hydrodynamics of the Czech Academy of Sciences, Pod Patankou 5/30, 16672 Prague, Czech Republic; peer@ih.cas.cz; 4 Faculty of Technology and Metallurgy, Ss. Cyril and Methodius University, Ruger Boskovic 16, 1000 Skopje, North Macedonia; jordanov@tmf.ukim.edu.mk; 5 Faculty of Science and Technology, Jan Długosz University in Częstochowa, 13/15 Armii Krajowej Av., 42-200 Częstochowa, Poland; p.rychter@ujd.edu.pl

**Keywords:** polymeric waste, waste management, circular economy loop, recycling, fine fibers, spinning

## Abstract

In modern society, it is impossible to imagine life without polymeric materials. However, managing the waste composed of these materials is one of the most significant environmental issues confronting us in the present day. Recycling polymeric waste is the most important action currently available to reduce environmental impacts worldwide and is one of the most dynamic areas in industry today. Utilizing this waste could not only benefit the environment but also promote sustainable development and circular economy management. In its program statement, the European Union has committed to support the use of sorted polymeric waste. This study reviews recent attempts to recycle this waste and convert it by alternative technologies into fine, nano-, and microscale fibers using electrospinning, blowing, melt, or centrifugal spinning. This review provides information regarding applying reprocessed fine fibers in various areas and a concrete approach to mitigate the threat of pollution caused by polymeric materials.

## 1. Introduction

Polymeric materials are inexpensive, lightweight, and durable materials processed into a variety of products that find use in a wide range of industrial and consumer applications. The undeniable benefits they brought to our society and economy have significantly increased their production [1], however, with the increase in consumption created by a growing world population and greater affluence amongst developing nations, the problems of a linear, resource-to-waste economy and environmental issues are becoming more acute [2]. Polymers that can be found in Nature (cellulose, lignin, chitosan, proteins, pectins, etc.) or synthesized in the industry as thermoplastics or thermosets have crucial importance in everyday life. Polymeric materials at the end of lifecycles and natural polymers as a by-product of agriculture, paper industry, fishing, or food production represent an enormous source of waste that must be seriously considered a source for new product lifecycle production. 

A linear economy system based on taking, making, using, disposing and polluting is responsible for millions of tons of waste that threaten people’s lives, while its littering and leakage in the environment cause negative impacts on land and sea life [3].

**Figure 1:**
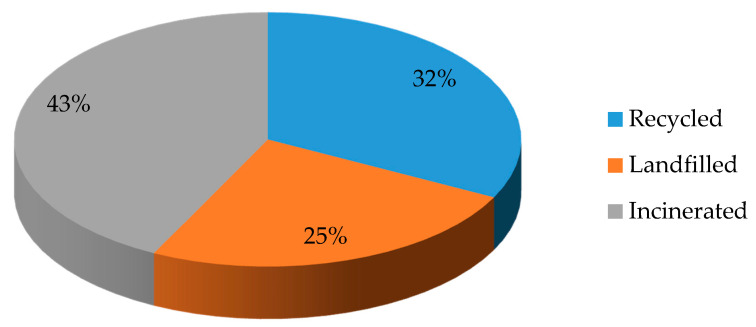
Management of the collected synthetic polymeric waste in Europe in 2018.

In 2018, an estimated 359 million tons of plastic (synthetic polymers) were produced globally. 29.1 million tons of them are collected in Europe. Ot these 32.5% are recycled, 24.9% are landfilled, and 42.6% are incinerated [4] (Figure 1).

**Figure 2:**
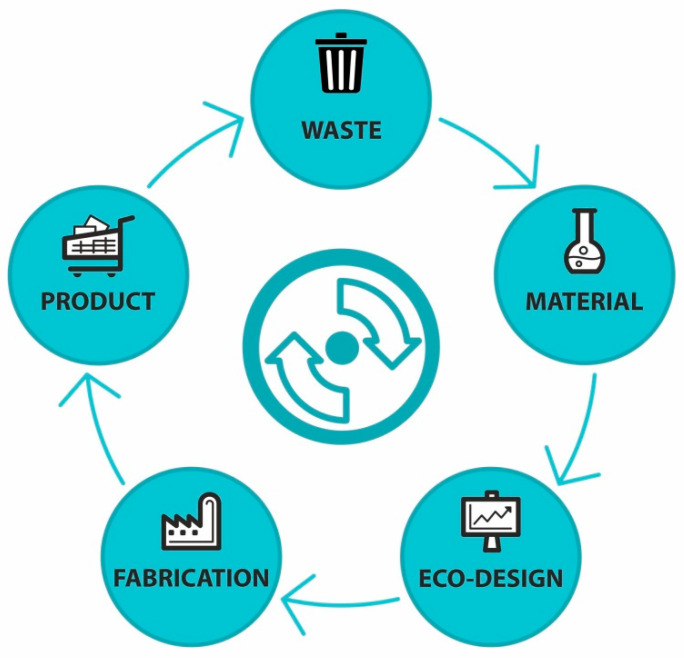
A simplified scheme of a circular economy in terms of material flow.

At present, the term “circular economy” is often discussed by experts and the general public in connection with plastics. The traditional linear economy model of production stands in contrast to the regenerative approach of the circular economy (Figure 2), which is a closed-loop product lifecycle with long-term targets to reduce landfilling and increase waste reuse and recycling [5,6]. Therefore, using polymeric waste as a source for renewable chemicals will be intensified in the future. However, closing the loop of material streams poses many challenges to research and innovation. Improving waste management and resource efficiency in industrial sectors, fostering industrial symbiosis, and remanufacturing waste are some of the many opportunities accepted by the research and industrial community. Even though the price of polymeric waste is low, the cost of recycling is not always economically viable, but consideration of the pollution of this waste in the environment overcomes the economic viability of recycling. The preferred options for recycling are obtained via mechanical or chemical pathways [7].

The circular economy is a combination of many initiatives [8]. Extending the product life, reuse, and recycling of the materials are unbreakable parts of the circular economy circle. Renewable waste as the starting and final point is the pivot of the circular economy model. The circular economy always looks for renewable wastes from production, agricultural waste, or other sources with strong renewable potential for the next product generation. Engineers must design the recycling of the product at the end of its lifecycle to produce the products based on the zero-waste concept.

The need for reuse is driven by the increasing use of traditional synthetic plastic products produced as a single use plastics made of synthetic polymers. As synthetic polymeric waste becomes a severe pollutant, many activities to make single-use (disposable) plastic reusable occur. When all possibilities to extend the lifecycle of the plastic through reuse are drained, the reprocessing (recycling) of the plastic waste into secondary products is a crucial option that stands well with a circular economy model. Recycling is the process that uses the waste to produce the same or products with less or added value. When the final products are added-value products, the process is named upcycling. Even though upcycling is more preferable, producing a new product with added value from renewable resources is not possible. In that case, we speak of downcycling, which produces less valuable recycled products. Plastic waste is often a mixture of various polymers with a high content of stabilizers, additives, and contaminants that can reduce the recycled products’ performance. Therefore, the complete separation of individual components is rarely implemented [8].

Reprocessing of polymers that is often accompanied by macromolecule degradation that leads to a reduction in viscosity, melt strength, and mechanical properties that has to be considered for proper recycling [9]. Nevertheless, plastic waste is already successfully used in many construction applications as a component in cementitious composites, asphalts, fillers, door panels, or insulation materials [10]. They are also used to prepare containers, agricultural pipes, compost bins, plastic tubes, food packaging, pallets, toys, disposable cutlery, drinking, detergent bottles, or carpet fibers [11]. Since electrospun nanofibers made from raw polymers have found great interest for biomedical purposes as a wound, especially burn dressing [12], in our opinion, more efforts should be put into investigating the usefulness of fibrous dressing wounds made from polymeric waste. Recent studies have demonstrated the alternatives of turning recycled polymers into fibers with diameters from hundreds of nanometers to tens micrometers [13,14,15]. Conventional textile fibers have a diameter from 4 to 100 µm, while microfibers are from 0.3 to 4 µm [16]. Even though nanomaterials are materials that have at least one dimension below 100 nm, in the fiber industry, fibers with a diameter of less than 1 µm are defined as nanofibers. With their high surface area to volume ratios, small pore size, and high porosity layers, nanofibers show excellent performance in air/liquid filtration, energy generation and storage, biomedical and tissue engineering, sensors, and catalysts, drug delivery, and nanocomposites. Their potential applications in various fields represent uses for recycling polymeric waste. To expand their use, polymer blends or the incorporation of additives into fibers can be applied.

The present state of knowledge deals with various types of polymer waste that can be reprocessed into fine fibers and used in a wide range of applications. Widely used worldwide fleece garments are among the best representative products commercially available from recycled PET [17,18]. Preparation of fibers from recycled PET in order to fabricate trendy fleece garments such as sweaters and jackets is one of the best examples of proper waste management based on upcycling.

Following the current pandemic situation all over and prognosis published up to date by WHO, there is no doubt that polymer fibrous membranes as masks against COVID 19 are currently one of the most demanded products ever [19]. With this respect, every alternative and environmentally friendly method of their fabrication should be seriously considered. The electrospinning method offers the opportunity to prepare fibrous polymer products from both not used and consumed polymers (plastic wastes). The randomly placed ultrafine fibers in the electrospun membranes are attractive as filtration materials. The high surface area to volume ratios, nano-porosity, good mechanical properties, and vapor permeability of such nanomembranes predestined them for air, water, or even personal protection against very fine dirt, bacteria, and viruses with dimensions smaller than 100 nm [20].

In this respect, there is an urgent need to combine, in one place, the new approaches connected with polymeric micro or nanofibers application in various fields of daily life. Therefore, the particular emphasis of this paper is focused the types of polymeric waste spinning process used to prepare micro and nanofibers and describe their application as well as advantages and disadvantages.

### Motivation

The disposal of polymeric waste poses severe problems because synthetic polymers are not biodegradable, while natural one can be easily recycled using new technologies that are still under examination. Landfill and incineration are reasonably cheap disposal methods, but these solutions are not environmentally friendly and have to be replaced. Nowadays, environmental problems have caused much interest in developing new biodegradable materials or using new recycling technologies to solve the increasing environmental burden problem [2,21]. Due to increasing concerns about preserving the environment and sustainability of resources, the extraction and the use of recycled polymer wastes is one of the alternatives to non-renewable resources in material technology [22]. The present state of knowledge deals with various types of polymeric waste that can be reprocessed into nano- and microfibers.

## 2. Types of Polymeric Waste

Based on its origin, polymeric waste may be derived from synthetic or natural sources generated from: (I) post-industrial waste and (II) post-consumer waste, which is an end-of-life product [7]. Polypropylene (PP), polyethylene (PE), poly(vinyl chloride) (PVC), polyethylene terephthalate (PET), polystyrene (PS), and polycarbonate (PC) are the most recycled synthetic polymers [23,24,25]. Recycling natural polymers and polymers from renewable resources such as cellulose, lignin, or poly(lactic acid) (PLA) has already been reported too.

**Figure 3:**
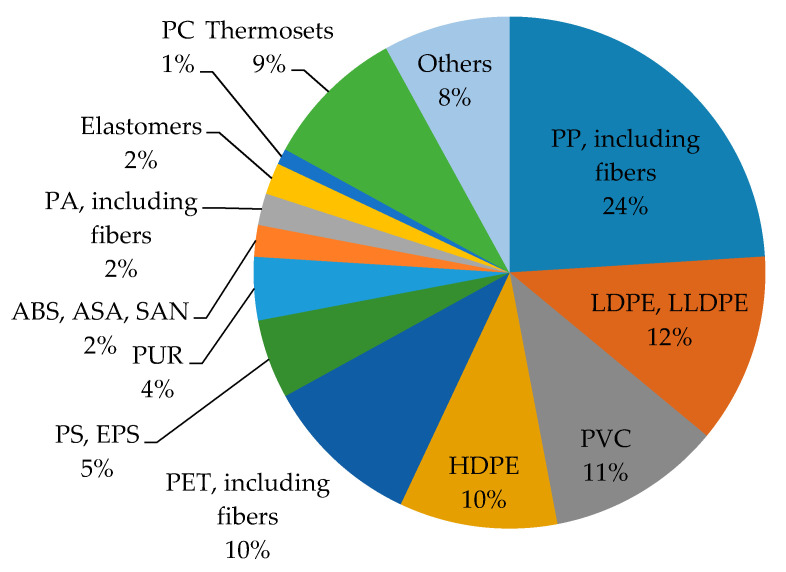
Percentage of globally-produced synthetic polymers in 2015.

In 2015, global synthetic polymer plastic production increased to 322 million tons (MT) [26] (Figure 3). Polyethylene, with 86.08 MT, represents 22% of total global plastic production. Low-density polyethylene (LDPE) and linear low-density polyethylene (LLDPE) comprise 12%, while high-density polyethylene (HDPE) accounts for 10% of all the produced plastic. With 61.87 million tons (16% plastic production), polypropylene is the second most-produced. PVC follows this leading group with 43.04 MT (11%), PET with 18.3 MT (5%), and other synthetic polymers amount to about 1% of global plastic production. Roughly 30% of PET is used in the food industry, including single-use plastic bottles. Similar environmental issues are caused the PE and PP as well. PE and PP are found in diverse fields such as packaging, textile, medicine, household furnishings and electronics [27]. Expanded polystyrene (EPS), known as styrofoam, is used for purposes such as insulation or packaging materials. Polycarbonate (PC) is another common synthetic plastic, especially for consumers of electronics in the form of light-emitting diode (LED) screens, smartphones, and blu-rays devices, as well as eyeglass lenses, kitchen utensils and household storage gear [28]. This plastic is dangerous because it contains bisphenol A (BPA), which leaches into the environment during its use and decomposition [29]. Poly(vinyl chloride) (PVC) is attractive from the recycling point of view because it is one of the most widely used plastics worldwide. Thanks to the physical and chemical advantage and its anti-degradable properties, and low production costs, PVC is widely applied as pipes, packaging materials, window frames, cables, bottles, and medical devices. Generally, the most common synthetic polymers can be used as feedstock for fiber production [30].

The recycling of conventional synthetic polymers is a more environmentally friendly process than sending a biopolymer to incinerated, composted, or dumped in a landfill. Among the main environmental benefits of polymeric recycling are energy conservation, saving depleting landfill space, and reducing petroleum use and CO_2_ emissions [31]. Even the end-of-life scenario of biopolymers is essential to determine real environmental sustainability. The opportunity of recycling polylactic acid (PLA), as a widely used biopolymer, undoubtedly represents a fascinating end-of-life solution [32,33]. In one available study polylactic acid used in the reprocessing technologies comes from food containers [34].

Lignocellulosic biomass comprised of cellulose, hemicellulose, and lignin is one of the most widespread biomass resources with strong potential for recycled fiber production [35]. With 170 billion tons [36,37], this biomass could be a serious pollutant if not appropriately treated. Cellulose is the most available, biodegradable, renewable biopolymer. It is also one of the most examined biopolymers, either pure or in derivative form. Like most natural bio-based polymers, cellulose does not melt but rather reshapes into continuous fibers upon dissolution. However, cellulose does not dissolve in common solvents because of its strong intramolecular hydrogen bonds. One of the most used paths for dissolving cellulose is via the viscose process by converting insoluble cellulose into soluble cellulose xantogenate by reaction with carbon disulfide in sodium hydroxide [38]. Other useful solutions for dissolving cellulose are dimethylsulfoxide/paraformaldehyde, sulfur dioxide [39], sodium hydroxide combined with urea and thiourea [40,41,42], inorganic metal complexes [43], molten inorganic salt hydrates [44], dimethylacetamide/lithium chloride mixture [45,46,47], *N*-methylmorpholine *N*-oxide, commonly known as NMMO [38], ionic liquids [48], etc. Some of these solvents are unsuitable for electrospinning applications, so researchers mostly study ether and ester-based cellulose derivatives for electrospinning [39]. Cellulose acetate (CA) is especially attractive due to its good solubility, and the fact it is commonly prepared from non-wood sources such as cotton waste [49,50]. Recently, despite the bad solubility of pure cellulose, ionic liquids could be a solution for processing cellulose into non-woven mats [51].

Lignin is the second biomass product after cellulose, with an annual growth rate of 10 billion tons [37]. It is only a renewable aromatic resource with a complex structure based on three basic phenolic monolignols that make up almost all types of lignin found in Nature [52]. Based on its origin, lignin exists as softwood and hardwood lignin [53]. As one of the biggest lignin producers, with 50 million tonnes annually, the paper industry produces lignin that is usually burned and used as a renewable energy source [36]. Based on extraction and isolation procedures, lignosulfonate, kraft, organosolv-lignin, and soda-lignin exists as available commercial lignins [37]. The botanical origin and extraction procedure determine a lignin’s solubility, molecular weight, and glass transition temperature [54]. The aromatic structure of lignin is promising for producing lignin-based carbon fibers and nanofibers. Traditionally, carbon fibers are made from polyacrylonitrile (PAN) fiber as a precursor that is a petroleum-based source, 5–10 times more expensive than a lignin-sourced precursor for carbon fiber production. Many explorations have been made to replace traditional PAN fiber with lignin-based fibers as a precursor for carbon fiber production. Aromatic structure, thermoplastic characters, solubility in different solvents, and low prices facilitate the production of lignin-based carbon fibers employing melt-pinning, wet-spinning, and dry-spinning [55].

Both types of polymeric waste, synthetic and natural, can be spun directly, blended with virgin or another polymer, or filled by doping with reinforcing additives.

## 3. Traditional Fiber Spinning

Several techniques for spinning fibers are summarized in [56,57]. Traditionally, fibers can be prepared from polymer melts or solutions. Melt spinning is the simple extrusion process suitable for thermally stable polymers. Solution spinning is applied for those polymers that cannot be melted. It can be divided into dry spinning (evaporation of solvent) and wet spinning (coagulation in a suitable liquid) [58]. The spinning of the polymeric waste directly is not always easy. It requires blending with the pure non-recycled polymers or adding the additives that will improve the final properties.

### 3.1. Direct Spinning of Recycled Plastics

Direct processing of polymeric waste is economically advantageous, but the resulting fibers may have inferior properties. Shin and Chase obtained expanded polystyrene (EPS) from a chemical bottle packaging without further purification [59]. Fine polymer fibers with an average diameter of around 700 nm were successfully electrospun from an EPS/D-limonene solution. Sow et al. used expanded polystyrene from shipment packaging, electronics packaging, laboratory chemicals packaging, disposable dishware, and craft polystyrene [60]. Fibers with a diameter less than a micrometer, useful for oil-water separation, have been produced from waste polystyrene dissolved in ethyl acetate by employing blowing spinning techniques. PET-based waste drinking bottles have been used for the fabrication of fine fibers with an average diameter in a range of 300 to 400 nm [61] while thinner recycled PET nanofibers (100 nm) were already obtained by electrospinning [20,62].

### 3.2. Spinning of Blended Recycled and Virgin Polymers

Blending recycled plastic with virgin polymer is one of the most frequent ways to produced fibers. Blending leads to reduce cost, while the properties of new blended fibers can still achieve outstanding performance. Bhat et al. [63] blended recycled and virgin polypropylene (PP) into fibers with micrometer diameters using melt-blown technology. They found that there is no need to blend more than 10–25% of recycled PP to maintain the properties of virgin PP. In the study of Tuladhar and Yin [64], melt-spun fibers with dimensions in the micrometer range were evaluated. The fibers were produced from a blend of 50% recycled PP and 50% of raw PP and are already commercially used due to their excellent mechanical properties. Lee et al. [65] used PET chips from water bottles and virgin fiber grade PET in various blends to fabricate microfibers using melt spinning. A 30/70 wt.% recycled/raw blended fibers exhibit similar mechanical properties to virgin PET fibers. Comparable results were observed in several other studies [66,67,68]. The spinning of a mixture consisting of different types of recycled plastic is very practical as it minimizes plastic sorting requirements. For instance, Zander et al. converted a mixture of three polymers into a fibrous layer [69]. Bottle-grade PET, PS, and PP were melt-electrospun in a ratio of 33/33/33 using the centrifugal spinning process into 1 to 12 µm fibers. These fibers were successfully applied for making ultra/microfiltration membranes, composites, and insulation. A blend of polycarbonate (from compact discs) and polystyrene (styrofoam) was dissolved in *N*,*N*-dimethylformamide (DMF) to be fabricated into fine fibers by electrospinning too. Enlarged fiber diameter with varied surface topography revealed the only partial miscibility of the polymers [14]. This examination has shown that 75/25 PC/PS recycled blend fibers produced by electrospinning are suitable for high-value applications like ultra/microfiltration membranes. Recycled PET from post-consumer soft drink bottles was used as an alternative reinforcing material for “in situ” microfibrillar–reinforced composite on the base of HDPE, and it was compared with liquid crystalline polymer (LCP) [70]. The r-PET/HDPE and LCP/HDPE composites were prepared by melt spinning at 190–250–255–260 °C and 190–220–220–225 °C temperatures extrusion zones. Styrene–(ethylene butylene)-styrene grafted maleic anhydride (SEBS-g-MA) was added into the blends for improved compatibility and processability. The diameter of the LCP/HDPE and PET/HDPE fibers was found to be between 5–10 µm, whereas the diameter of r-PET fibers ranged from 0.5 to 10 µm. The obtained thermal stability results demonstrated the high potential of r-PET in replacing the more expensive LCP to improve the thermal resistance of HDPE. Moreover, the r-PET component played the role of a well-defined reinforcing component for HDPE-based thermoplastic composite, in the presence of at least 5 wt.% of SEBS-g-MA.

Telli and Ozil tested the properties of knitted fabrics from the r-PETfibers prepared by melt spinning [71]. They proved that fabric produced with r-PET fibers does not have the same properties as fabric made from PET fibers. However, these fibers can be blended in the apparel industry with primary raw materials such as cotton and PES without noticeable changes in the textiles’ quality. By adding 30% of r-PET fibers into cotton, higher bursting strength can be obtained, while blending a small amount of r-PET with PET will not comprise the fabric performance. Moreover, the economic impact of blending recycling and virgin polymers is significant because the apparel price decreases. After all, r-PET is about 20% cheaper than cotton and PET fibers.

### 3.3. Filler Reinforcement

Recently, several investigations have focused on improving the properties of recycled fibers by using fillers. Sisal fibers have been used as reinforcing filler in fibrous composites produced from r-PET by exploiting to prepare materials with a high added value [72]. The presence of cellulose in nanocomposites creates superhydrophilic surfaces. Khan et al. [73] used PS from a local restaurant to produce fibers incorporating multi-walled carbon nanotubes (MWCNTs) and NiZn ferrite nanoparticles. These fillers increased the thermal conductivity, hydrophobicity and surface roughness. In another research work, Mohammadkhani et al. [74] modified r-PET fibers’ surfaces with magnetic nanoparticles. The saturation magnetization, wettability, and electromagnetic interface shielding of composite mats were improved compared to a rPET mat.

Lignin can be used as a fiber structure additive too. Its aromatic structure shows charring capability to be a good flame suppressant and inhibitor of flammable drops during the burning of synthetic fibers [37,75]. Polylactic acid melt spun fiber with lignin shows promising flame retardancy, too [76].

## 4. Alternatives to Traditional Spinning Technologies

In addition to melt-spinning, as one of the most common technologies, alternative technologies for nano- and microfiber production, which are well known for researchers but not commonly used in the industry, are described in this review. The methods below are most frequently used to form nano- and microfibrous mats from the recycled polymers.

**Figure 4:**
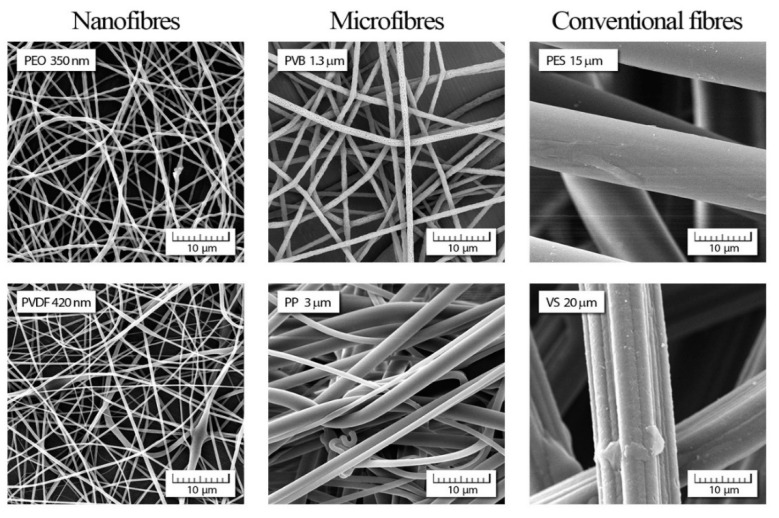
SEM images of the nanofibers (polyethylene oxide (PEO); polyvinylidene fluoride (PVDF)) compared to the microfibers (polyvinyl butyral (PVB); polypropylene (PP)) and the conventional fibers (polyester (PES); viscose (VS)). Source: private archive of the authors.

Generally, the fibrous mats acquire advantageous characteristics by decreasing the fibers’ diameter (Figure 4). Their large specific surface area and the highly porous 3D structures are useful in many application areas, wherein previous decades they had no chance, namely, as filtration, protective clothing, and biomedical applications.

### 4.1. Electrospinning

**Figure 5:**
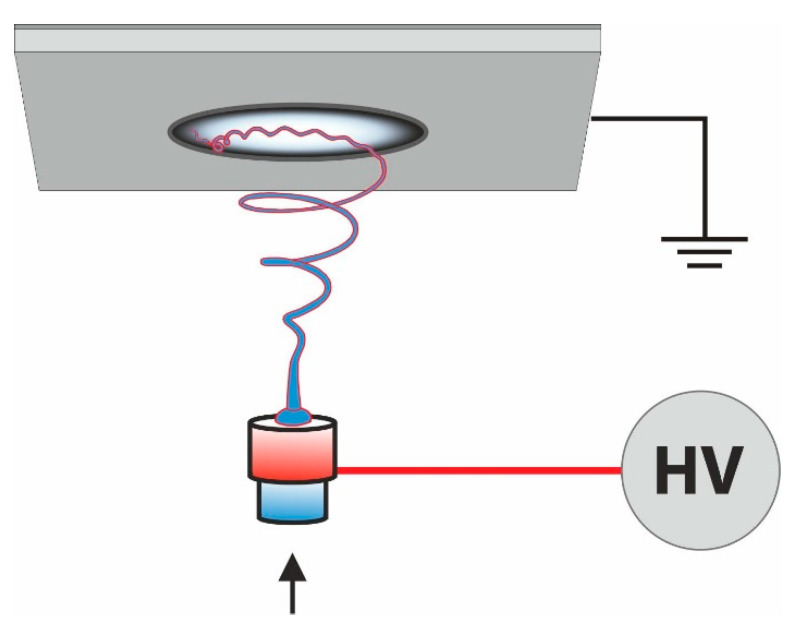
Sketch of an electrospinning apparatus.

Electrospinning is a technique to produce fine fibers from polymer solutions or melts where the fibers are spun in a high electric field. The polymer drop is deformed into an apex (Taylor cone) at the spinneret (syringe, pipette, wire, rotating cylinder) due to this high electric field. The polymer jet is drawn, elongated, and the dried fibers are collected on a grounded flat or rotating collector. The process is displayed in Figure 5. The fiber morphology is influenced by parameters which can divided roughly into several basic groups: polymer properties (solubility, molar mass), solvent properties (boiling point, volatility), polymer solution properties (concentration, viscosity, conductivity), the process parameters (applied voltage, top of the needle to collector distance, flow rate, needle diameter or cylinder rate), and environmental parameters (relative humidity and temperature) [77,78]. Recently, various electrospinning technique modifications have been developed, such as needless electrospinning, multiple-jet electrospinning, bubble electrospinning, cylindrical porous hollow tube electrospinning, electro-blowing, coaxial, and charge injection electrospinning [79,80,81]. Electrospinning has several advantages: (I) it can be used for more than 200 different polymers [82]; (II) fibers can be functionalized before, during, and after spinning; (III) low cost and commercial availability for industrial production; (IV) fibrous layers can be deposited onto a variety of substrates [79].

Nevertheless, electrospinning has also some limitations: (I) some polymers lack an appropriate solvent; (II) evaporation of the solvent changes the fiber surface; in many cases, the solvents used are toxic, and the evaporation process can be lengthy; (III) the capillary can be blocked [81] and (IV) low productivity of conventional needle electrospinning. However, this problem can be overcome by using a multi-nozzle system [22]. The system was tested for mass production of stacked styrofoam fine fibers. The waste styrofoam was dissolved in the mixture of tetrahydrofuran (THF), citronella, and cajuput oil. The produced fine fibers exhibited the same quality as those produced by conventional electrospinning.

Electrospinning is a very attractive technology, mostly due to the properties offered by the fabricated materials. Conventional solutions or melt electrospinning have already been used for the recycling of plastic waste.

Gomes et al. concluded that fibrous membranes could be obtained from recycled PET via the electrospinning process [21]. In this study, the 1,1,1,3,3,3-hexafluoro-2-propanol (HFIP) was used as a solvent. It was shown that electrospinning is an excellent alternative for processing recycled PET and to obtain material consisting of fine fibers (ranging from 187 to 936 nm) with high functional value. Moreover, even after processing, the PET fibers retained their properties without showing signs of degradation.

Esmaeli et al. [83] prepared mat-like fibers from bottle-grade PET (a mixture of TFA/DCM was used as solvent) from styrofoams, PS, and PC (the last two polymers using DCM as solvent) from compact discs by solution electrospinning. The authors investigated the effects of electrospinning processing parameters such as applied voltage in the range of 15–25 kV or dimensions of the needle (0.23 and 0.69 mm) on the diameter and distribution of all three types of polymers from plastic waste. As a result, it could be generalized that the increase of the applied voltage led to a rise of the mean fiber diameter. However, the fibers’ diameter distribution is narrowest at the voltage of 20 kV. The lower the needle diameter, the lower the average diameter of PET fibers (179 nm), and their narrowest size distribution was observed. The same tendency during the electrospinning process was noticed for PC and PS fibers (average diameter 164 and 213 nm, respectively); however, the broadest fibers diameters distribution was observed for the PS sample.

Abbas et al. examined polymer concentration, needle tip to collector distance, feed rate, and applied voltage on the morphology of fibers obtained during a PET electrospinning process [84]. The polymer concentration in TFA/DCM in ratio 1/3 was found as the most influential factor for the fiber diameter. The optimal electrospinning parameters for PET from packaging waste were: 5% polymer solution, 1 mL·h^−1^ feed rate, 15 cm needle tip to collector distance, and 15kV applied voltage were found to give an average diameter of 105 ± 37 nm.

Polyvinyl chloride was collected from pipe waste in the study of Antonakou and Achilias [28]. The formation and morphology of fibers were observed after electrospinning in alternatives solvents, in DMF, tetrahydrofuran (THF), and dimethylacetamide (DMAc). The relationship between concentration (5, 10, 15, and 20 wt.%) and morphology was investigated in this study. During electrospinning the process parameters such as flow rate, applied voltage, and distance from the collector to the needle tip were kept constant. From the microscope images, seen a critical concentration of PVC solution in which electrospun PVC there has been experienced a morphological change from particles to fibers. The most acceptable beads-free mats were obtained from THF solution with a concentration of 20 wt.%. The average diameter of the fibers was 2.83 ± 1.46 µm. Concerning the reprocessing of cellulose waste, some authors have reported the preparation of cellulose acetate membranes by electrospinning as well.

Han et al. studied the effect of solvent composition on the fiber diameter [85]. A mixed acetic acid/water solvent system in ratio 75/25% for CA fine fibers’ electrospinning was evaluated in the study. Uniform CA fine fibers with an average diameter of 160 nm were electrospun from a 17 wt.% CA solution. The average diameters of the CA fine fibers could be controlled between 160 nm to 1280 nm by changing the mixed solvent composition from 75/25% to 95/5% acetic acid/water, respectively. The nonwoven mat structure of the CA fibers was well maintained during their subsequent deacetylation. The first production of lignin-based nanofibers was made using a lignin spinning solution in ethanol (lignin: ethanol = 1:1). Ethanol evaporates rapidly and hence forms a solid supernatant. Several solvents, including DMF, DMSO, and water, have been utilized to dissolve lignins. As the most common solvent for solution preparation due to its excellent solubilization of lignin, DMF showed useful applications [86]. Researchers have utilized water, which is the cheapest and most environmentally friendly solvent as an alternative to organic solvents. It was found that when alkaline water is applied as a solvent, the resulting lignin nanofibers have improved thermal stability [55].

Melt electrospinning is more environmentally friendly than the conventional solution electrospinning method because it does not use any harmful or toxic solvents. Still, there is another challenge due to its difficulty in melting r-PET. The effect of melting temperature of r-PET on fiber diameters was studied by Naksuwan et al. [87] by using the melt-electrospinning method. The melted r-PET granules at a temperature of 260, 290, and 310 °C, were electrospun at a high voltage of 38 kV, and the distance between the die to the collector of 12 cm. The average diameters of the obtained electrospun fiber, which is melted at melting temperature of 260 °C, 290 °C, and 310 °C, were 65.74 µm, 45.18 µm, and 60.32 µm, respectively. From this result can be concluded that the melting temperature impacts the diameter of the resulting fibers.

### 4.2. Solution Blowing Spinning

**Figure 6:**
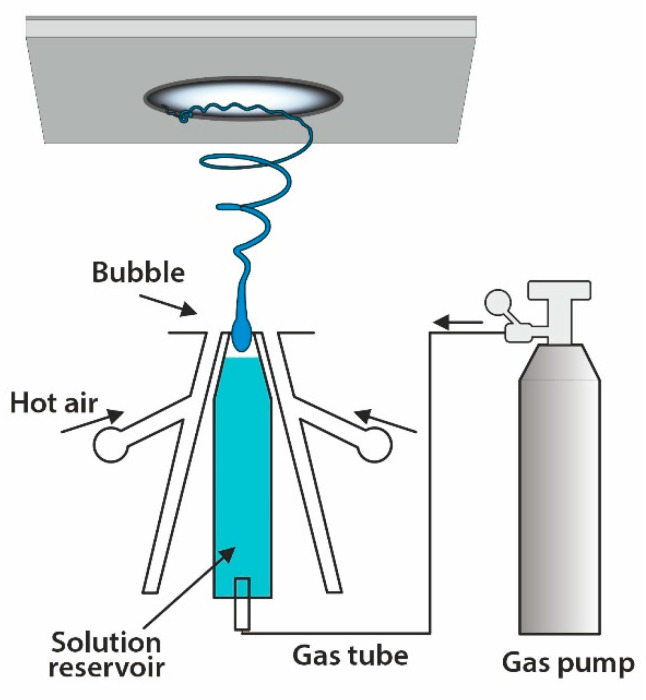
Sketch of a solution blow spinning apparatus.

Solution blow spinning has emerged as a rapid and straightforward technique to produce nano- to micrometer diameter fibers using a pressurized gas to drive the fiber formation [88]. This process requires a polymer solution and a pressurized gas that flows around, so the created fibers are deposited in the gas flow direction. The method is displayed in Figure 6. The fiber formation is affected by the solution properties, while the operating parameters govern the fibrous texture. The main parameters include polymer type, solvent, polymer solution concentration, the working distance (nozzle-to target distance), and gas pressure [61]. The main advantages are: (I) fiber production is 10 times faster than electrospinning [89]; (II) it does not require high electrical potential and conducting targets; (III) it can be utilized for in situ deposition of fiber scaffolds [90]. The mechanical properties of fibers are inferior compared to electrospun fine fibers [91]. The mechanical properties of PCL fabricated by solution blowing spinning and electrospinning have been compared. It was shown that mats prepared by blowing spinning had a lower modulus (2 ± 1 MPa) than electrospun PCL (13 ± 1 MPa). The stress of electrospun PCL was 3 ± 0.5 MPa compared to 1.25 ± 0.25 MPa. The higher entanglement of individual electrospun PCL nanofibers most likely caused them to have higher stiffness than nanofibers prepared by blowing spinning, which was more loosely packed and less entangled [91].

Singhal et al. experimentally demonstrated the fabrication of waste expanded polystyrene (WPS) fine fibers using solution blow spinning [2]. The selection of solvent is critical from the economic and environmental points of view. The effect of three solvents, with distinct volatility, ethyl acetate (EA), toluene (To), and DMF on the fiber formation has been studied and the ease of the solvent recovery examined in order to ascertain the practicability of using these solvents for the recycling process. Comparison of the presence of To, DMF, and EA demonstrated the preparation of uniform fibers with only a few beads and smallest diameters in the following order: To (222 ± 115 nm) > DMF (613 ± 151 nm) > EA (635 ± 336 nm). Also, the authors conclude that by reducing the temperature of the condenser outlet to 0 °C, it was possible to recover ~73 wt.% of EA, ~80 wt.% of To, and ~77 wt.% of DMF. The recovery of solvents has a positive effect on the economic and environmental aspects of the research.

### 4.3. Melt Spinning

**Figure 7:**
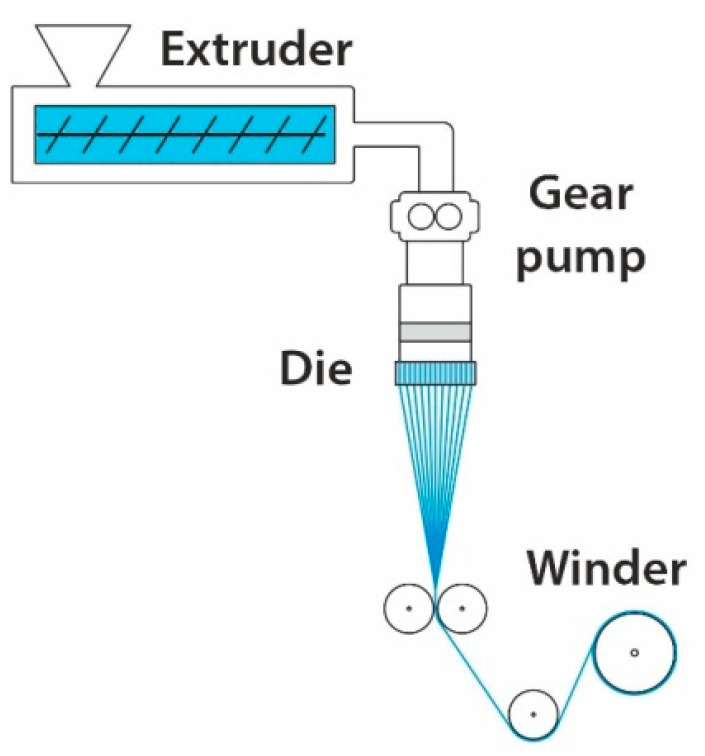
Sketch of a melt spinning apparatus.

Melt spinning is a standard technique for fiber formation in the polymer industry. During this process, polymer pellets are heated to the proper temperature and extruded through micron-sized dies (orifices), and then rapidly cooled [92] (see Figure 7). This technique produces fibers with a diameter in the order of a few hundred micrometers; nevertheless, it can have several modifications in the cooling zone using air, such as melt-blown spinning. The melt-blowing process uses a high-velocity air jet to rapidly elongate a fine fiber with a submicron diameter [93]. The reduction of diameter depends on using the highest possible airflow rates, the diameter of orifices, spinning temperature, and extrusion rate [94]. There are many advantages of melt spinning, such as: (I) high production efficiency; (II) low cost and easy to implement large scale production; (III) ability to create controlled cross-sections of fibers (star shape, hollow and grooved fibers) [92]. The disadvantages are polymer thermal degradation and the impossibility of incorporating nanoparticles (metal, ceramic, carbon) into the polymer melt.

Jiang et al. prepared and tested recycled PET (r-PET) fibers made from PET waste by using the melt spinning method, and the results were compared with the original PET (o-PET) [95]. The spinning temperature for both types of polymers was set at 290 °C. The average diameter of both r-PET fibers and o-PET fibers was around 19.5 µm. The r-PET fibers demonstrate a greater breaking strength, smaller elongation at break resulting from their lower crystallinity, and higher degree of orientation. The thermal stability of r-PET is significantly lower as a result of impurities during the recycling processes.

Regarding chemical structure as confirmed by FTIR, the r-PET and o-PET have the same structures. r-PET was also studied by Abbassi et al. [96]. The influence of a high spinning speed 3000 and 3500 m·min^−1^ and the spinning temperature 280, 285, and 290 °C on the fibers’ properties were studied. The increase in the rate improved the density, crystal size, tenacity amorphous, and crystal orientation of r-PET filament. The study of viscosity showed that the recycling process resulted in decreasing the molecular weight of polymers. The crystallinity, crystal size, tensile, and intrinsic viscosity values decreased at higher spinning temperatures, but the shrinkage increased. The average diameter observed by SEM in investigated samples was around 20 µm. This study confirmed r-PET as the right candidate for use in melt spinning processes.

Due to the expensive and difficulties at conventional recycling methods of PLA (depolymerization), Tavanaie studied the production of PLA fibers from PLA food packaging waste by a melt spinning method [34]. In this study, melt-spun mats prepared from pre-dried recycled poly(lactic) acid (r-PLA) flakes just before spinning with an average diameter of 31 µm and with a linear density of 0.91 mg·m^−1^ demonstrated better mechanical properties. The initial modulus was affected by the molecular chain orientation and crystallinity of the fiber. According to the differential scanning calorimetry results, the best structure of r-PLA fiber was obtained from the samples spun at 180 °C. A further advantage of this material was that fibers have excellent dye uptake at the dyeing temperature of 110 °C. Bishal et al. [97] used the melt spinning process for biodegradability modification of polyamide 6 (PA 6) by r-PLA from plastic food containers. The blends PA 6 and PLA with composition from 5 up to 40 wt.% of PLA were tested. The authors reported that the addition of 5 and 10 wt.% of PLA in PA6/PLA blends significantly improved their mechanical properties. Further increasing PLA concentration in tested blends was not beneficial from a processing point of view, and the mechanical properties of materials only decreased. Nevertheless, biodegradability enhancement of PA 6/r-PLA fibers with increasing the r-PLA content was confirmed. 

Tavanaie and Mahmudi [98] studied the possibility of modifying PP fibers via blending with r-PLA by a melt spinning method using temperatures of the barrel and die zones up to 190 °C. The results showed that the PP/r-PLA mixture fibers with different blend ratios could be successfully melt spun with suitable continuity. The average diameters of melt-spun fibers ranged between 260 and 470 nm according to the content of r-PLA in the sample. Good tenacity, initial modulus, and suitable biodegradability were obtained for the modified PP fibers doped with 40 and 50 wt.% of r-PLA dispersed phase. The biodegradability of the blend fiber sample with 50% of r-PLA content was about 36.2%. Dye uptake of the modified PP fiber was enhanced, and the washing and lightfastness of some of them were excellent.

The constantly growing consumption of plastics bags worldwide leads to problems related to their management after use. Most plastic bags are made from low density or high-density polyethylene (LDPE or HDPE). The thermal technology to produce fuel for disposing of such waste is energy-intensive. Therefore, Tutak et al. discussed recycling plastic bag waste into textile fibers by melt spinning at a temperature of 147 °C [91]. The authors reported the characteristics of recycled fibers such as linear density, crystallinity, tenacity, and melt spinning parameters’ effect on the features. Fiber diameter can be manipulated by changing the take-up axial velocity; higher take-up axial velocity leads to lower fiber count. Higher take-up axial velocity influences the increment of the tensile strength of the recycled fiber. The smaller linear density of fiber leads to higher fiber tenacity. Recycled polyethylene fibers have a great potential application in non-apparel textiles [99].

### 4.4. Centrifugal Spinning

**Figure 8:**
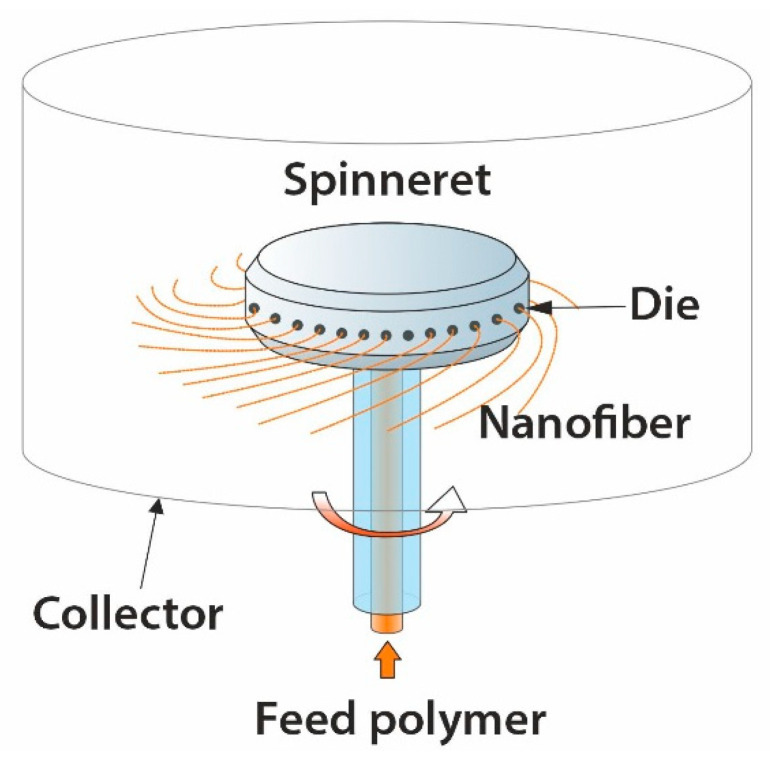
Sketch of a centrifugal spinning apparatus.

Centrifugal spinning is known in the two versions; first, it uses a heated spinneret that melts and elongates the polymer jet using centrifugal force, enabling nano- to the micro-fiber formation [100]. The sketch of this apparatus is shown in Figure 8. The second variant of this method used a polymer solution instead of a melt [101,102]. The fiber formation depends on the competition between the polymer jet’s viscous forces, surface tension, and applied centrifugal forces. The main process parameters include angular speed, viscosity, and surface tension, die radius and direction, chamber temperature, and distance to the collector [93,103].

Centrifugal spinning has several significant benefits: (I) fiber production is 50 times higher than electrospinning [103]; (II) it can work with viscous polymer melts or polymer solutions; (III) both conductive and non-conductive materials can be used. However, the design and selection of heating devices, temperature measurement, and control systems are rather complicated [93].

Vo et al. used the centrifugal spinning of a r-PET solution in the fabrication of fibers ranging from nanometers to micrometers in scale from a mixture of trifluoroacetic acid and dichloromethane [102]. The influence of the polymer solution concentration, rotational speed of the spinneret, inner diameter of the needles on the formation, morphology, and mechanical properties of the fibers was examined. A 10 wt.% concentration and a rotation speed of 15,000 rpm appeared to be the most appropriate conditions to produce bead-free fibrous membranes with an average fiber diameter of 619 ± 235 nm and with strength 4.3 MPa and modulus 34.4 MPa.

Paganotto et al. produced recycled fibers from EPS dissolved in chloroform through centrifugal spinning. They evaluated the effects of the rotational speed and concentration of the polymer solution parameters. The morphology and diameter of the formed fibers were investigated according to the operating parameters [104]. The results showed that an increase of polymer solution concentration generates a higher average diameter. Concentrations of 5 *w*/*w*%, 10 *w*/*w*%, and 15 *w*/*w*% have been tested, and average diameters 410 nm, 1120 nm, and 15 μm has been obtained, respectively. However, in less concentrated solutions, continuous fibers have been obtained, promoting the higher production of beads. The evaluation of rotational speed shows that the higher rotational speed, the lower the fiber diameter. A diameter of 9460 nm has been obtained with a rotational speed of 5000 rpm. On the other hand, 1280 nm has been achieved with a rotational speed of 15,000 rpm. The characteristics of the different spinning methods as well as their advantages and disadvantages are summarized in Table 1.

## 5. Applications of Fibers from Polymeric Waste

Fibrous materials prepared from recycled feedstocks via one of the techniques mentioned above could be used in various applications. Fine fibers may be successfully used for ultra- or micro-filtration membranes [105] or applications such as technical textiles [106], construction materials [66], catalysts [107], or medicine [108].

### 5.1. Filter Applications

Expanded polystyrene (EPS) is one of the plastics most recycled into the form of micro or ultrafine fibers by conventional electrospinning for effective filter media. An air filter was developed by Rajak et al. by recycling expanded polystyrene (r-EPS) waste to be repurposed as fine EPS fibrous mats using electrospinning [109]. The highest capture efficiency was achieved almost 100% by the fiber with the smallest diameter (314 nm), and the lowest efficiency was achieved at 70.4% by fiber with the largest diameter (3500 nm). The efficiency was relatively high compared to the filter from fine fibers fabricated by Liu et al. from virgin polymers [110]. Also, the quality factor of r-EPS fibrous mats was better than fibers prepared from virgin polymers by Zhang et al. [111].

Water-in-oil emulsion separations are essential to the petrochemical industry for product quality, safety, ecologic and economic reasons. This was the motivation for Shin et al. [112], who prepared fine fibers with a diameter of around 600 nm from EPS waste. The fibers’ addition having a diameter less than one micrometer to conventional micron-sized fibrous filter media improved the filter media’s separation efficiency from 68 to 88%. Shin et al. [113] also developed a composite membrane to separate water-in-oil emulsions with a drop size of less than 100 µm. The composite membrane consisted of micro glass fibers and fine fibers electrospun from r-EPS. The experimental results showed that adding fine fibers to conventional glass filtration media improves the filter media’s separation efficiency. An optimum performance occurred with 4% of PS fibers (diameter 500 nm) by mass to glass fibers for the filters.

Ezzatzedeh et al. worked on preparing filters using recycled PS foam from food containers enriched by magnetic nanoparticles Fe_3_O_4_ for nitrate removal from water [114]. For obtaining recycled polystyrene, the food containers were chopped into small pieces and dissolved in N,N-dimethylformamide (DMF) to form a homogeneous polymer solution of 40% concentration. Then, synthesized magnetic nanoparticles were added and stirred for 24 h till a uniform dispersion was obtained. Different concentrations of 2, 11, and 20% of Fe_3_O_4_ by polymer weight in such filters were evaluated. During the electrospinning process, the solution was fed to the tip at a flow rate of 20 µL min^−1^. A positive voltage of 20 kV was applied to the polymer solution. By optimizing the significant electrospinning parameters, uniform polystyrene- Fe_3_O_4_ composite fine fibers with diameters in the range of 50 to 300 nm were prepared. Nitrate removal was conducted on magnetic fine fiber composites via adsorption. Increasing the amount of magnetic nanoparticles can lead to better nitrate removal efficiency.

Fine fibers fabricated by Zander et al. from waste PET were tested as liquid filtration membranes [62]. Fiber diameters as low as 100 nm were achieved; the mats were unsuitable for ultrafiltration applications since particles under 500 nm were not effectively removed. However, the mats could be used in microfiltration applications such as a pre-filter in a wastewater treatment system. A supporting layer will likely be needed due to the fibers’ relatively low tensile strength (ca. 5 MPa) unless used in very low-pressure filtration applications. Polyethylene terephthalate is typically “down-cycled” into lower-value products, such as clothing fabrics, so converting it into higher-value filtration membranes could provide a strong economic incentive to improve recycling rates Pulido et al. [115].

Tough fibrous membranes for smoke filtration have been developed from recycled PET bottles by Strain et al. using solution electrospinning as fabrication technology [116]. The effect of trifluoroacetic acid and dichloromethane solution concentration in ratio 70/30 wt.% on the morphology was observed. The 10 wt.% concentration, 7 kV applied voltage, and 5 µL·min^−1^ appeared to be the most appropriate parameters due to the smallest diameter 410 ± 120 nm, the largest specific surface area (7.07 m^2^·g^−1^), and it also contained the highest amount of absorbed smoke components (43.7 times its weight). With increasing polymer solution concentration, the average diameter of the fibers increased even up to 4.3 µm; the specific surface area decreased (below 0.67 m^2^·g^−1^) together with the real mass uptake smoke components. The high affinity of these r-PET fiber mats for airborne hydrocarbons also opens the way for their applied use in a range of industrial filters.

Opálková Šišková et al. examined the alternative use of PET bottle grade waste for personal protection purposes during the coronavirus pandemic [20]. A PET facial mask consists of a free-standing membrane with a thickness of 25 µm, with a basis weight of 14 g·m^−2^. The average diameter of the fibers present in the membrane was 95 ± 37 nm, prepared by electrospinning from the HFIP/DCM solution. Measurements revealed high filtration efficiency (more than 98%), to capture particles with dimensions of 120 nm. The vapor permeability was 94%, and the membrane’s breathability is at the lower limit of the comfortable range. Still, it could be improved by modifying the filter thickness, filter area, and filter shape. Protective masks are stretched in all directions during the application; therefore, the mechanical properties of the free-standing membrane of r-PET have also been tested as one of the crucial parameters of the protective material. It was found that the tensile strength was 2.7 ± 0.3 MPa, and the average elongation to the break was 46 ± 9%. The results have been compared to the values obtained from testing the materials used for surgical masks from 1-layer polypropylene nonwoven fabric prepared by melt-blown technology with a basis weight of 28 g·m^−2^ and 2-layers of cotton fabric, with a basis weight 148 g·m^−2^ used by people from domestic sources during a pandemic. The filtration efficiency of r-PET membranes was incomparably more significant.

Opálková Šišková et al. [117] investigated the fibrous free-standing composite membranes. On the basis of a previous study [20], electrospun membranes composed of r-PET made from bottle waste and silk fibroin (SF) extracted from the cocoons of silkworm (*Bombyx mori*) have been prepared from the solution in HFIP/DCM in the ratio 30/80% (*v*/*v*). Silk fibroin should improve the air permeability and decrease the pressure drop to enhance the user comfort properties. The effect of the amount of SF on the filtration effectivity and air permeability has been studied. Membranes with basis weights in the range of 10–12 g·m^−2^ exhibit 43–99% filtration effectivity (FE) and membranes with basis weight in the range 1.7–3.2 g·m^−2^ exhibit FE values of 39–96%. The membrane r-PET with 54.5% of SF had a FE 90.23%, and pressure drop of 59 Pa. Such a membrane has been classified as FFP1 type according to the EN149 standard. The membranes with lower amounts of SF exhibited too high pressure drop, while membranes with higher SF exhibit good air permeability but very low FE. On the other hand, the effectivity of tested membranes r-PET, r-PET/16.6.% of SF, and r-PET/54.5% SF classified them as class E11, E10, and E10, respectively, in the case of lower basis weight and into the class H13, E12, and E12 in the case of higher basis weight according to EN1822.

Moreover, the antibacterial activity has been tested, and the viability of two strains of bacteria, *S. aureus* and *E. coli*, was reduced by around 95% after 24 h of contact time. The biocompatibility of investigated membranes has been proven as well. The studied membranes r-PET and r-PET/54.5% SF were shown to have a non-cytotoxic effect on keratinocytes after 48 h of incubation compared to control.

The objective of the study of Bonfim et al. [118] was to produce filter media with microfibers obtained from PET bottles waste for the highly efficient removal of nanoparticles present in the air. Electrospinning has been used as a fabrication technique. Trifluoroacetic acid (TFA) and DCM in the ratio of 70/30% have been used as solvent. The developed filter media with an average fiber diameter of 1.29 ± 0.84 μm proved to be an excellent air filter to capture the nanoparticles with a high collection efficiency of 98.4%, with a low pressure drop of 212 Pa. In addition, the filter media had good mechanical properties (4 MPa). The filter media can be used in environmental remediation and personal protection, cleanroom, and indoor air purification. Attia et al. [119] investigated the preparation and application of electrospun membranes composed of lignin, successfully extracted from palm fronds and banana bunches as biomass sources using the organosolv fractionation technique, and PET from bottle waste which enabled the production of fibers with smooth morphology for the adsorption of methylene blue dye (MB) from the water. The fibers with the diameter 1800 ± 500 nm have been obtained from the polymer solution HFIP/DCM ratio of 2:1 with the final concentration of 24.5%. The strength of the membrane has been about 3.2 MPa, and elongation at the break is about 69%. The membrane has been treated with iodine and carbonized at 500 °C to obtain the carbon fibers (CFs). The achieved carbon content did not exceed 62 wt.%. The adsorption capacity of CFs was tested, and it was reported about 9 mg of MB/g CFs. The work demonstrates the potential of combining biomass waste as a lignin source with sustainable waste (PET) to produce fibers as a core material that can be used for environmental remediation.

Nonwoven mats fabricated by one of the mentioned alternative techniques are predestined for filtration application by their morphology which is given by the randomly placed fine fibers, and by their high specific surface area, porosity, and small sized pores, or by good air permeability.

### 5.2. Other Promising Applications

The literature on using nano and microfibers fabricated using the above-mentioned alternative techniques in other applications such as filtration is limited. However, the existing literature gives the scientists and people from the industry a signal to consider the new application possibilities and expand the processing of polymer wastes. Among such promising applications there are building, construction, catalyst, or even medicinal uses.

The production and use of recycled polypropylene (PP) fibers are limited. However, Tuladhar and Yin focused on recycling PP fibers’ industrial-scale production by mechanical recycling [64]. The recycling of PP fibers from industrial wastes was performed by melt spinning at temperatures ranging between 218–235 °C. The recycled PP fibers with dimensions in micrometers exhibited superior mechanical properties. The tensile strength of 100% recycled PP fibers was 340 MPa, and Young’s modulus was 7100 MPa. To compare the results, tensile strength 435 MPa and 9000 MPa Young’s modulus of fibers was achieved by blending 50% recycled PP and 50% of virgin PP, which was comparable with the mechanical properties of 100% virgin PP fibers (450 MPa of tensile strength and 7500 MPa of Young’s modulus). The 100% recycled PP fibers produced in this study are now commercially available as “Emesh”. Such recycled fibers have already been used to reinforce concrete on footpaths, carparks, and precast elements to replace traditionally used steel mesh in buildings and constructions.

Traditional heavy and corroding steel reinforcements can be replaced by polyacrylonitrile (PAN)/lignin-based carbon fiber composites as a new promising material class for the building industry [120]. These materials have higher protection of the environment, a longer lifecycle, and, thus, cause much less damage to structural components of the concrete materials used in the building and construction field.

Several authors have reported the recycling of PET by melt electrospinning. In the study of Rajabinejad et al., recycled PET was melted at 200–235 °C and electrospun with an applied voltage at 25 kV [106]. The effect of melt spinning parameters, including flow rates, the distance between nozzle and collector, on the morphology and finesses of produced fibers were investigated. The range 61–93 nm diameter has been achieved when 0.1 mL·min^−1^ and 3–5 cm spinning distance were set. Melt electrospinning was considered an appropriate and environmentally friendly way of recycling post-consumer bottle grade PET. It can prevent more pollution of the environment, which is the main advantage of this technology compared to solution electrospinning.

Bottle grade PET waste (BG) and raw PET fibers (FG) were recycled by Gurudatt et al. by melt spinning at an extrusion temperature of 260 –300 °C and a drawing temperature up to 200 °C [66]. The blending effect of FG and BG in various ratios (100/0, 75/25, 25/75, and 0/100 (wt.%)) on the fiber properties was studied. The impact of the draw ratio on the fibers was evaluated as well, and it was found that with increasing draw ratio, the linear density decreased. The linear density of undrawn fiber was 132 den, but the linear density of fibers drawn by the rate of 5.8 was 21.8 den. Authors reported that bottle-grade PET possesses a unique combination of high strength and extensibility. The most exciting observation was that the stress at break of fibers spun from 100% PET BG waste polymer was nearly 1.2 times higher than those produced from 100% virgin FG. The authors blended BG with raw FG, and their blending improved the melt processing and the properties of fibers produced from it. Such fibers could be used in traditional applications and technical textile applications previously unknown for recycled fibers.

Electrospun nanofiber-based on recycled expanded polystyrene (EPS) shows excellent potential in the thermal insulation field to restore historic buildings in the narrow central parts of the old towns [121]. Expanded PS was dissolved in DMF and DMF/DCM (ratio 1:3) in the concentration 15% (*w*/*v*), respectively. The manufactured nanofiber mats were macroscopically homogenous and showed excellent insulation properties, e.g., very low thermal conductivity. The lowest thermal conductivity (20 mW·mK^−^1) was shown by the EPS membranes from a blend of the DMF/DCM solvents with fiber diameters of 1.8 μm, due to the lowest porosity (82%). The decrease of porosity in EPS in DMF with a fiber diameter of 3.4 μm (80%) leads to higher thermal conductivity (24 mW·mK^−1^). 

Nanofibrous mats can serve as catalysts in environmental remediation applications. The purification of water from harmful organic compounds like bacteria, viruses, or even textile dyes has become a serious concern. A composite based on TiO_2_/styrofoam membranes prepared using electrospinning was developed by Rajak et al. [122] to prevent a water hygiene crisis. Styrofoam from waste was used for the study, and TiO_2_ as semiconductor catalyst material used in the environmentally friendly photocatalysis process was incorporated into the fine styrofoam fibers. The particles of TiO_2_ have high photocatalytic activity because of their vast range of light absorption, and by reducing the size of TiO_2,_ the photocatalytic activity is increased. The observations of such fibrous composites’ photocatalytic activities showed that the generated fine fiber does not hinder TiO_2_ particles from performing photocatalytic processes.

In the research of Yasin et al. [107], PET nanofibers were produced by electrospinning waste PET polymer. In the next step, CuO nanoparticles have been crosslinked on the surface of the electrospun PET nanofibers to enhance the photodegradation of the dye methylene blue and adsorption of the degradation products. The study revealed that an eco-friendly, cheap, sustainable, and effective nanocomposite can be produced using this process, and the efficiency of the dye removal is above 99% after 30 min.

Nowadays, there is a growing interest in the application of nano and microfibers in medicine. In a study of Grumezescu et al., electrospun nanostructured fibrillary membranes based on r-PET and silver nanoparticles were used for antimicrobial applications [108]. The membranes were prepared by electrospinning from the blend of TFA and DCM. To obtain fibrous r-PET material with antimicrobial properties, silver nanoparticles (varying between 25 and 85 nm) were applied on the surface of the r-PET fibrous membrane (fibers with diameters ranging between 60 and 250 nm). The r-PET fibrous covered by silver nanoparticles demonstrated good antimicrobial and antibiofilm activity against Gram-positive and Gram-negative bacteria as well as against fungal strains. Moreover, tests showed also lower cytotoxic and inflammatory effects. With this great advantage, such material could be further implemented in the biomedical coating area for implants, medical surfaces, and textiles.

Cellulose acetate present in cigarette butts was regenerated by Hemamalini et al. into fibrous mats [49]. Mats have been prepared by electrospinning from a blend of acetone (AC) and DMF in the ratio 2:1. The spinning process, conducted at voltage 20 kV, was optimized. Bead-free membranes with a fiber diameter in the range between 200–700 nm was obtained from a solution with a concentration of 12% (*w*/*v*). For potential application of the CA electrospun membranes, the solution was doped with silver nitrate; however, it was only useful in low concentrations because a high amount of (AgNO_3_) caused an undesirable increase in solution conductivity and viscosity is not appropriate for the electrospinning process. The authors found that the addition of silver nitrate was optimal up to 10% (*w*/*v*), and for this reason, solutions were doped by 1, 3, and 5 mM of AgNO_3_. The concentration affected the uniformity of the fibers; the mat demonstrated good antimicrobial activity. The zone of inhibition is found to be higher for Gram-negative (*S. aureus*) bacteria as compared to Gram-positive bacteria (*E. coli*). An effective way for utilization of the disposed of cigarette butts was shown in this study.

As described above, fibers applications made from polymeric wastes confirm their sophisticated utilization in various fields like filtration technology, medicine, or technical textiles. Fibers, especially reused ones, are not only essential ingredients to enhance product quality and improve people’s lives, but they can also help preserve our planet´s natural resources.

It is especially important to reuse plastic waste in textile production because growing consumption of these products causes significant environmental, climate, and social impacts by using resources, water, land, and chemicals, emitting greenhouse gases and pollutants [123]. Although recycling polymer waste converts this material into a reusable one, the quality of the recycled fibers depends on the type of polymer and the recycling process used. One of the methods to maintain the required material property is by mixing with virgin fibers. Recycled fibers of polyesters, polyamide, cotton, or wool have already been successfully used to produce professional wear; however, polyesters are of the greatest interest in this area. Considering the fact that sustainability drives innovation, there are more and more developing companies focusing on the utilization of polymer recyclates contributing to the consumption of environmentally harmful and persistent wastes, especially microplastics generated during polymer degradation [124]. They help to set future trends and give possibilities for the future application of these fibrous materials. The addition of polymer fibers may be found in professional and luxury textiles and clothes (carpets, backpacks, tents, antimicrobial, moisture resistance, thermal comfort, easily stretched work, and sports clothes) [125,126]. Moreover, fibers have found application in hygiene products, flushable cleaning wipes, sustainable packaging, or battery separators.

The usefulness of recycled polymers as matrices in glass fiber-reinforced composites as well as improvement of concrete properties is also in an extensive research and application phase [127,128,129]. For such applications, composite properties are dominated by the reinforcement (glass fibers), and with this respect even polymer recyclates without compatibilization may provide the composites with satisfactory mechanical properties. Approaches using fibers made from waste poly(ethylene terephthalate) as a concrete reinforcement have demonstrated some drawbacks due to hydrolysis of the ester linkages of PET in the highly alkaline environment of the cement matrix. To overcome the problem of alkaline hydrolysis of this polymer, the PET fibers were coated with ethylene/vinyl acetate copolymer (EVA). The authors demonstrated that the introduction of the PET fibers does not deteriorate the mechanical strength of the concrete composite; however, due to worsening in workability of the concrete mix, the addition of PET fibers only up to 0.3% (*v*/*v*) is recommended.

Numerous industrial applications of fibers both taken from raw materials, natural or synthesized and made from polymer waste as recyclates, confirm the great interest in these materials among environmentally responsible companies worldwide. Growing awareness of the ecological aspects of polymer waste inspires producers to be innovative and simultaneously sustainable production of second life goods, meeting the expectations of social and environmental demand.

## 6. Conclusions

Growing worldwide attention on polymeric waste leads to searching for new ways towards their environmentally safe utilization. A European strategy for polymeric waste use in the future focuses on designing and producing polymeric materials, which can be reused or recycled. For this reason, there is an urgent need to promote polymeric waste after its use. This review aims to map “alternative” technologies to recycle polymeric waste and prepare nano/microfibers for various applications in everyday life. The further promising utilization of fine fibers composed by one of the previously discussed methods is being sought. Difficult to stop, still growing contamination caused by polymer waste, including microplastics, is the subject of tightening up legislation by the European Union related to waste management. Although landfill is a relatively cheap disposal route, it is still the last preferred waste management option compared to reuse and recycling of polymer waste [130].

Consistently nondegradable polymers (PP, PET, PE, PVC, EPS, and PC) dominate in landfill sites, possessing economic and environmental concerns to society. For this reason, it is worth considering these materials as a representative waste of resources for the second use. Although the recycling process of these polymer materials involves pollution and energy consumption, it is still, after reuse, the best way to minimize the negative impact on the environment. Since total recycling of plastics is not possible, mechanical, chemical, biological processing of polymer waste, even additional energy consumption, seems to be the only way to reduce still growing landfill sites emitting contaminants into the air, water, and soil all over the world. Growing consumption of biodegradable polymers worldwide gives hope for the reduction of polymer contamination. Nevertheless, due to cost, insufficient sources, and difficulty in processing, these materials cannot successfully replace conventional plastics in the present.

This study focused on using polymer wastes to prepare fibers because global production and consumption of fiber-made materials have constantly increased in past decades, especially in medicine, filtration processes, construction, the automobile, and energy sectors (fiber-reinforced polymers). Despite the traditional fiber spinning, the electrospinning technique providing nano and micro-fibers has become increasingly popular in medicine or filtration. Since the production of fine fibers possesses some disadvantages, many efforts are being put to overcome these problems because both micro and nanofiber materials become currently in great interest of various industrial branches, especially when used from polymer wastes, leading to limitation of plastic environment pollution.

Upcycling plastics waste into high-value products for various applications is indeed a sustainable solution with a promising future. It allows for the conversion of post-consumer products and can help alleviate the burden of solid wastes on the environment. Moreover, the realization of recycling is highly multidisciplinary. Partnerships among fundamental science, material, engineering, and processes development are essential for integrating existing manufacturing methods.

Fortunately, increasing legislation related to plastic waste management improves the mismanagement of plastic waste in developed countries. However, middle and low-income countries are becoming the main sources of global plastic pollution, possessing poor or no plastic waste management systems resulting in up to 90% of polymer waste being improperly disposed [131].

The environmental responsibility of both country authorities and each company producing and providing polymer products on the market, including strategy related to waste management, should be the main challenge in light of environmentally sustainable development objectives.

## Figures and Tables

**Table 1:** Characteristics of different spinning methods.

Type of Spinning		Advantages	Disadvantages	Literature
Melt spinning	Dry spinning	High production efficiency; Low cost and easy large scale production; Ability to create fibers with controlled cross-sections (star shape, hollow and grooved fibers).	Thermal degradation of the polymer; Impossibility of incorporating nanoparticles (metal, ceramic, carbon) into the polymer melt.	[92]
Solution spinning	Dry spinning (evaporation of solvent)	It can be used for any polymer; Fibre can attain strength comparable with maximum theoretical strength; The process can be continuous.	The production rate is low; One or more bath is required for completely removal of the solvent from the polymer (for wet spinning); Post-spinning operations are more length; High costly production. The formation of exact fibre cross section is difficult to control because of inward and outward mass transfer process (especially for wet spinning).	[58]
	Wet spinning (coagulation in a suitable liquid)			
Electrospinning needless electrospinning multiple-jet electrospinning bubble electrospinning cylindrical porous hollow tube electrospinning electro-blowing, coaxial electrospinning charge injection electrospinning	Solution	Used for more than 200 different polymers; Functionalization of the fibers before, during, and after spinning; Low cost; Available for industrial production; Fibrous layers can be deposited onto a variety of substrates.	Some polymers do not have an appropriate solvent; Evaporation of the solvent changes the fiber surface; The used solvents are often toxic; The evaporation process can be lengthy; The capillary can be blocked; Low productivity of conventional needle electrospinning.	[79,80,81,82]
	Melt	More environmentally friendly than solution electrospinning; Does not use any harmful or toxic solvents.	Difficulty in melting	
Solution blowing electrospinning	Polymer solution and pressurized gas	Fiber production is 10 times faster than electrospinning [89]; It does not require high electrical potential and conducting target; Utilized for in situ deposition of fiber scaffolds.	Inferior mechanical properties of the fibers	[89,90,91]
Centrifugal spinning	With heated spinneret that uses polymer melts	Fiber production is 50 times higher than electrospinning; It can work with viscous polymer melts or polymer solutions; Both conductive and non-conductive material can be used.	Complicated control system for heating devices	[93,100,103]
	With spinneret that uses polymer solution			[101,102]
